# Risk Assessment of *Brucella* Exposure Through Raw Milk Consumption in India: One Health Implications and Control Strategies

**DOI:** 10.3390/vetsci12050465

**Published:** 2025-05-13

**Authors:** Vijay Sharma, Balbir B. Singh, Victoria J. Brookes

**Affiliations:** 1 Centre for One Health, Guru Angad Dev Veterinary and Animal Sciences University, Ludhiana 141004, India; vijaysharmajammu2@gmail.com (V.S.); bbsdhaliwal@gadvasu.in (B.B.S.); 2 Sydney School of Veterinary Science, Faculty of Science, The University of Sydney, Camperdown, NSW 2006, Australia; 3 Sydney Infectious Diseases Institute, Faculty of Medicine and Health, Westmead, NSW 2145, Australia

**Keywords:** *Brucella abortus*, bovine, cattle, India, pasteurisation, Punjab, risk assessment

## Abstract

Brucellosis is a zoonotic disease with significant public health implications. Understanding the risks of consuming unpasteurized (raw) milk is critical for effective control measures. A quantitative risk assessment was conducted to estimate *Brucella abortus* contamination in milk from unregulated sources in Punjab, India, where 70% of milk is sold unpasteurized. Samples from lactating cattle and buffalo (N = 261) in ten villages were tested using the Rose Bengal plate test and indirect IgG ELISA. Modelled risk pathways estimated *B. abortus* shedding probabilities and colony-forming unit (CFU) concentrations in milk, with Sobol sensitivity analysis identifying influential parameters. Buffalo had a higher estimated shedding prevalence (0.04, 95% PI: 0.02–0.07) than cattle (6.3 × 10^−3^, 95% PI: 2.5 × 10^−3^–13.2 × 10^−3^). Mean contamination levels were 2843 CFU/100 mL (95% PI: 0–32,693 CFU/100 mL) for cattle, 17,963 CFU/100 mL (95% PI: 612–67,121 CFU/100 mL) for buffalo, and 7587 CFU/100 mL (95% PI: 82–39,038 CFU/100 mL) combined. High-shedding animals were the most influential factor (total effect sensitivity index of 0.86 [95% CI: 0.63–0.74]). Removing high-shedding animals reduced risk considerably for people who might drink raw milk once (absolute risk reduction of up to 54% in buffalo milk), but once-per-month consumption is still likely high risk. Effective risk mitigation requires a One Health approach, strengthening both public and animal health interventions, because animal health strategies alone will fail if milk from high-shedding animals reaches the unregulated milk market.

## Simple Summary

Brucellosis is a serious veterinary and public health concern in India. Given that drinking milk is an important transmission risk for brucellosis in people, we investigated the risk of *Brucella abortus* exposure from milk sold via the unregulated supply chain in Punjab, India, to identify the influential factors that could be targeted by control measures. We estimated that even 100 mL of raw milk from unregulated sources is likely to contain *Brucella abortus* colony-forming units that exceed 1000 CFU/100 mL, presenting an alarming public health risk from a single meal of 100 mL unpasteurized milk. High-shedding animals were found to have most influence on risk, and whilst their removal could reduce the risk on a single occasion, consumption of 100 mL with as low of a frequency as once monthly posed a considerable risk. It is clear, therefore, that without the elimination of *B. abortus* in cattle, an ongoing One Health approach incorporating consumer education with regard to boiling milk, improved regulation of milk sales, and animal health measures (vaccination of cattle, and identification and removal of high shedders) are required. These measures are also not without considerable challenges, highlighting that without strong advocacy, brucellosis will remain a neglected disease of vulnerable people.

## 1. Introduction

Brucellosis is a zoonotic disease caused by bacteria of the *Brucella* genus. It has recently been estimated that at least 1.6–2.1 million new cases of human brucellosis occur every year, predominantly in low- and middle-income countries where the disease remains endemic in livestock [1]. Symptoms include undulating fever (also a colloquial name for brucellosis, in addition to Mediterranean or Malta fever), weakness, fever, myocarditis, arthritis, neuropathies, orchitis, and urethritis [2].

Among *Brucella* species that affect terrestrial animals, *B. abortus*, *B. melitensis*, and *B. Suis* (biovars 1 and 3) cause brucellosis in cattle; of these, *B. abortus* is the most frequently reported [3]. Brucellosis is endemic in bovine populations in Punjab, India [4]. A recent meta-analysis estimated a countrywide combined prevalence of brucellosis in cattle and buffalo of 16.6% (95% confidence interval [CI] 13.0–21.1%) and 14.2% (95% CI: 8.9, 21.8), respectively [5]. In non-pregnant cattle, the infection is usually asymptomatic, but in pregnant cattle it can cause placentitis, resulting in pregnancy loss in late gestation, with shedding of the organism in placental, fetal, and vaginal fluids, as well as in milk due to infection of the mammary gland [6]. Whilst infected animals might only abort once in their lifetime, they can remain infected and shed *Brucella* organisms indefinitely [7].

As well as large economic losses in livestock in India [8], dairy farmers and veterinary personnel are particularly at risk of acquiring brucellosis [9]. People in these occupational roles can be infected by direct contact with aborted fetuses and related materials; however, the most common route of infection is through consumption of raw milk or other unpasteurized dairy products [10]. Therefore, the burden of brucellosis and the socioeconomic impact in the broader public is also high, with an estimated loss of 177,601 DALYs (disability-adjusted life years; 95% UI 152,695–214,764) at a rate of 0.29 DALYs/1000 people/year (95% UI 0.08–0.70) in occupational and 0.13 DALYs/1000 people/year (95% UI 0.06–0.18) in non-occupational adult populations [11].

Punjab, India is an agrarian state, home to over 6.5 million dairy cattle (*Bos taurus*) and Murrah buffalo, a breed of water buffalo (*Bubalis bubalis* [12]). The per capita milk availability is higher here (1245 gms/day) than other Indian states, and the total milk production was 140,001,800 tonnes in 2023–24 [12]. Of this, only 30% was sold by regulated suppliers who pasteurize milk, with the remaining 70% sold as raw milk by unregulated suppliers such as informal milk shops and bicycle vendors [13], demonstrating the strength of this market and a potentially high risk of *Brucella* exposure to people via this route.

Globally, there are numerous studies of risk factors for *Brucella* infection in bovines and people, yet very few risk assessments. In bovines, risk factor studies have ranged across investigations of the factors associated with seroprevalence in buffalo (for example, it has been found to be higher in buffalo kept in wetlands than on dry land, and in female than male buffalo [14]), and animal and herd-level risk factors in cattle (for example, higher seroprevalence has been associated with risk factors such as free-grazing farming in Uganda [15], and the presence of calving paddocks in Brazil [16]). A recent meta-analysis of risk factors in people determined a far greater odds of brucellosis in people who consumed unpasteurized milk (odds ratio 3.86; 95% CI 1.81–8.23). It is surprising that despite the high global burden of brucellosis in people, very few risk assessments have investigated the pathways that lead to infection in people and potential control points. Examples of risk assessments in the context of *Brucella* include a quantitative assessment of transboundary pathways into Great Britain [17], a qualitative assessment of the exposure of consumers in Sudan to *Brucella* in milk [18], and a quantitative assessment of risk of human brucellosis infection through informally marketed milk in Kampala, Uganda [19].

Given that drinking milk is an important transmission risk for brucellosis infection in people, we investigated the risk of *Brucella abortus* exposure from milk produced and sold via the unregulated supply chain in Punjab, India, to identify the factors that could be targeted by control measures. Empirical data were limited; therefore, methods and input parameters were supported by global studies and a comprehensive sensitivity analysis was conducted to identify key influential parameters.

## 2. Materials and Methods

A quantitative risk assessment was conducted to estimate the predicted probability that milk consumed from unregulated sources in Punjab, India, would contain an infectious dose of *B. abortus*, and the probability of human infection given the consumption of 100 mL of unpasteurized milk on a single occasion or once monthly for one year. Unregulated sources include informal milk shops or bicycle vendors from which milk can be consumed unpasteurized, as well as milk consumed by producers in their own households. Regulated sources sell pasteurized milk; therefore, exposure to *Brucella* via this route does not occur. Model outputs were the expected distribution of CFU/100 mL milk sold by unregulated sources in two scenarios based on differing shedding probabilities in *B. abortus* seropositive dairy cows and buffalo. Model outputs also included the probability of human infection following the consumption of 100 mL once, or 100 mL once monthly for one year. The infectious dose of *B. abortus* by inhalation for people has been estimated at 10–100 colony forming units (CFU; Corbel [20]); therefore, due to lack of data on oral infection with *B. abortus*, an infectious dose increased by an order of magnitude (1000 CFU orally) was used. Although national guidelines recommend an intake of at least 300 g/person/day of milk in India, we elected a more conservative consumption of 100 mL/person/day.

### 2.1. Model Parameters

#### 2.1.1. Brucella Seroprevalence in Lactating Cattle and Buffalo

The seroprevalence of lactating cows and buffaloes was investigated in the study region. Ten villages (seven rural and three peri-urban) in Ludhiana district of Punjab were randomly selected. The sample size of farms/village was calculated using a probability proportional to size approach (number of farm households) based on an expected animal-level *B. abortus* prevalence of 50%. The farm households (N = 84) were randomly selected from a sampling frame of farm households in each village. All lactating cattle and buffalo were blood-sampled on each farm (median 3; range 1–13). Approximately 5–10 mL blood was taken from the jugular vein of each animal and left to clot. Samples were transported in cool boxes to the laboratory at the Guru Angad Dev Veterinary and Animal Sciences University (GADVASU), Ludhiana, Punjab, and kept at 4 °C. Serum was separated by centrifugation of the clotted blood at 3000 rpm for 15 min. The collected serum was aliquoted in screw-capped sterilized serum vials and stored at −20 °C until testing.

Collected sera (N = 261) were tested for *B. abortus* antibody using the Rose Bengal plate test (RBPT) and indirect ELISA (I-ELISA; Table 1). Overall, 46 and 58 of the lactating animals (cows and buffalo) were positive using the Rose Bengal plate test (RBPT) and indirect ELISA (i-ELISA), respectively. Of all the tested animals, 41 animals were positive based on both the RBPT and i-ELISA. Stratified by species, of 181 cows, 35 and 45 were positive using RBPT and i-ELISA, respectively, with 33 cows positive based on both the RBPT and i-ELISA, and of 80 buffalo, 11 and 13 were positive using RBPT and i-ELISA, respectively, with 8 buffalo positive based on both the RBPT and i-ELISA.

The number of cattle that were positive in both tests (as the most conservative estimate) was used to determine the apparent seroprevalence according to a beta distribution (Table 2). The overall seroprevalence, as well as cattle-specific and buffalo specific apparent *B. abortus* seroprevalences based on a beta distribution were 0.16, 0.19, and 0.11 (95% ranges 0.12–0.21, 0.13–0.25, and 0.05–0.19; Table 2). Based on evaluation of the performance of the same serological tests in cattle in Bangladesh [21], the diagnostic sensitivity (Se) and specificity (Sp) of these tests used in the current study were considered as follows: RBPT Se = 87.4% and Sp = 99.4%, and i-ELISA Se = 84.6% and Sp = 99.6%. The diagnostic Se and Sp for the tests used in series was calculated as 0.7927 and 1 respectively, according to Equations (1) and (2) in which Se (Se) and Sp (Sp) in series are functions of the combined test (T) characteristics. True prevalence was simulated using Equation (3) [22] to give an estimated *B. abortus* true seroprevalence overall, and by species (*Seroprevalence_true_*).

(1) $${Se}_{series}={Se}_{T1}*\;{Se}_{T2}$$

(2) $${Sp}_{series}=1-\left(1-{Sp}_{T1}\right)*(1-{Sp}_{T2})$$

(3) $${Seroprevalence}_{true}=\frac{{Seroprevalence}_{apparent}+(Sp-1)}{Sp+(Se-1)}$$

#### 2.1.2. Probability of Brucella Abortus Shed in Milk of Seropositive Cattle and Buffalo

The probability that a seropositive cow or buffalo would shed *B. abortus* CFUs in milk depends on the stage of infection, the individual’s status, such as a recent abortion or the stage of lactation, and the individual’s immune response. In a study conducted in Italy [23] in which *B. abortus* seroprevalence and prevalence in milk was investigated in 500 water buffalo [23], *B. abortus* was detected in milk by culture (considered a gold standard test by study authors) in 30% (101) water buffalo of 337 buffalo that were seropositive by RBPT (the least specific test used in the study; therefore, the most conservative proportion for the current study). In contrast, a study in Iran identified *Brucella abortus* in 8.6% (57/675) of milk specimens collected from seropositive cows in Alborz province and *B. abortus* (*n* = 6) and *B. melitensis* (*n* = 11) in 6.5% (or 2.3% and 4.2% *B. abortus* and *B. melitensis*, respectively) of 260 milk specimens collected from seropositive cows in Tehran province in which cows were seropositive by RBPT, a serum agglutination test, a 2-mercaptoethanol test, i-ELISA and a milk ring test [24].

Therefore, a beta distribution with mean 0.30 (95% CI 0.25–0.35; Table 2) was used to describe the highest probability of shedding based on water buffalo in Italy, and a beta distribution of mean 0.03 (95% CI 0.01–0.05; Table 2) described the most conservative probability based on shedding in cows in Tehran, Iran.

The prevalence of shedding (*Prev_shed_*) was calculated according to Equation (4), according to each scenario (buffalo [Italy] and cows [Iran]) and the overall prevalence, given the proportion of seropositive cattle and buffalo in the sample from Punjab (assuming each species shed according to the scenarios identified in the studies from Italy and Iran, respectively).

(4) $${Prev}_{shed}={Seroprevalence}_{true}*\;Pshed$$

#### 2.1.3. Number of *B. abortus* Colony Forming Units/mL Milk

Few studies have investigated the number of *B. abortus* CFUs shed in milk. In the study conducted in Italy [23], 73 buffalo were consistently low shedders in milk (≤1000 CFU/mL), 16 animals were high shedders (≥10,000 CFU/mL milk), and 12 animals were intermittent low shedders (≤1000 colony forming units (CFU/mL). The *B. abortus* detection threshold in the study was 30 CFU/mL; therefore, we made the assumption that intermittent low shedders were animals which were sometimes in the range 1–29 CFU/mL (i.e., shedding below the detection threshold), giving a total of 85 buffalo shedding 1–1000 CFU/mL milk. The highest level of shedding was derived from an experimental study in which *B. abortus* was detected in concentrations as high as 4 × 10^4^ CFU/mL in milk samples [25]. Based on these studies, we estimated the proportion of low shedders (*RLowShed*; uniform distribution minimum 1 CFU/mL, maximum 1 × 10^3^ CFU/mL of milk) of lactating cattle and buffalo that were shedding *B. abortus* in milk in Punjab as mean 84% (95% range 77–90%) based on a beta distribution (*PLowShed*; Table 2). The proportion of high shedders (*RHighShed*; uniform distribution minimum 1 × 10^4^ CFU/mL, maximum 4 × 10^4^ CFU/mL of milk) was estimated as 1 − *PLowShed*.

Based on previous studies [25], we made the assumption that the number of *B. abortus* CFU/mL would be stable in the milk given that the time from collection to consumption of milk from unregulated sources (informal milk shops or bicycle vendors) would be short (most frequently <1 day). Raw milk is mixed on farm (if from >1 animal), then sold to unregulated milk vendors who mix it into 20–40 L aluminum or plastic cans (local knowledge: *pers. comm.* Dr. Jaswinder Singh, Professor and Head, Department of Veterinary Animal Husbandry & Extension Education, GADVASU, Ludhiana, India). Therefore, we estimated *B. abortus* CFU/mL of homogenously mixed milk from the farms in the current study, to determine if this reached sufficient dose to infect a person.

Previous studies have reported economic losses due to *Brucella* infection in cattle, including lower milk production. In a study in Gujarat, India, Panchasara et al. [26] reported an average loss of 10% of total lactation yield in seropositive cows and buffalo. Therefore, assuming homogenous mixing and a 10% lower proportion of milk from seropositive animals, the mean number of *B. abortus* CFU/mL milk from shedding cows and buffalos (*CFU*/*mL_shed_*) and from all cows and buffalo (*CFU*/*mL_all_*) was estimated according to Equation (5) and Equation (6), respectively. The CFU/100 mL of milk was then simulated by random sampling from the distribution of *CFU/mL*_100_.

(5) $$CFU/{mL}_{shed}=(PLowShed*RLowShed)+((1-PLowShed)*RHighShed))$$

(6) $$CFU/{mL}_{100}=CFU/{mL}_{shed}*\;100(\frac{{Prev}_{shed}*0.9}{{(Prev}_{shed}*0.9)+(1-{Prev}_{shed})})$$

Of 107 milk consumers who bought milk from unregulated sources in Punjab, India, 11 (10.3%) reported that they consume raw milk (*pers. comm*; *K. Singh 2022*). Three people consumed raw milk daily, 5 people consumed milk occasionally (1–2 times weekly), and 3 people consumed milk rarely (<1/year). Therefore, given a hypothetical infectious dose (ID_50_) of 1000 CFU orally, the probability of human infection (*P*(*infection*)) was estimated for the consumption of the CFU in 100 mL raw milk for the three scenarios (Equation (7)), as well as the annual probability of infection given consumption of 100 mL once monthly (*P*(*annual infection*); Equation (8)), recognizing that this is a conservative estimate for some consumers who buy milk from unregulated sources and consume it raw.

(7) $$P\left(infection\right)=1-e^{-CFU/{ID}_{50}}$$

(8) $$P(annual\;infection)=1-(1-{P(infection))}^{n}$$

A variance-based global sensitivity analysis was implemented using the Sobol’ method in the ‘sensitivity’ package in R [27]. This evaluated the influence of input parameters on *B. abortus* CFU/mL milk. The influence of the 95% range of each input distribution on output variance was estimated, both with (total effect sensitivity indices [SIs]) and without (main effect SIs) its interactions with other inputs. Main and total effect SIs were plotted as centipede plots with 95% confidence intervals.

## 3. Results

**Figure 1:**
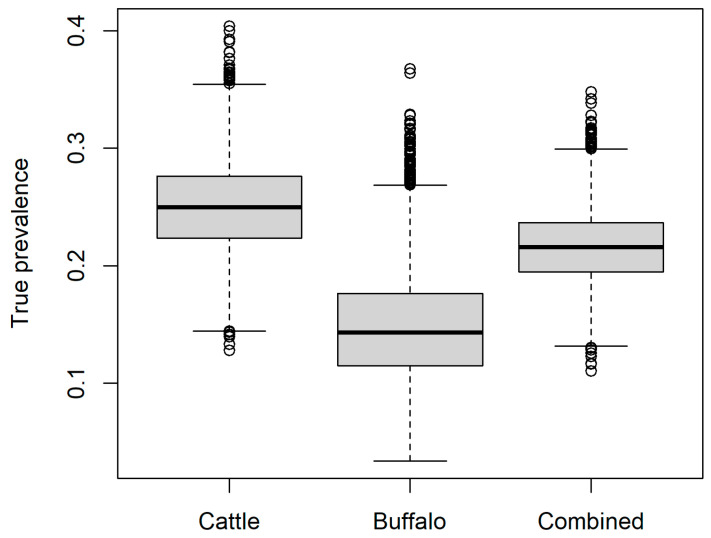
Estimated true seroprevalence of *Brucella abortus* in the current study for buffalo (*Bubalis bubalis*), cattle *(Bos taurus*), and as a combined group according to the proportions of each species sampled in the current study.

The estimated true *B. abortus* seroprevalence in the study population overall (all animals) was mean 0.21 (95% range 0.16–0.28), with mean 0.25 (95% range 0.18–0.33) and mean 0.15 (95% range 0.07–0.25) for cows and buffalo, respectively (Figure 1).

**Figure 2:**
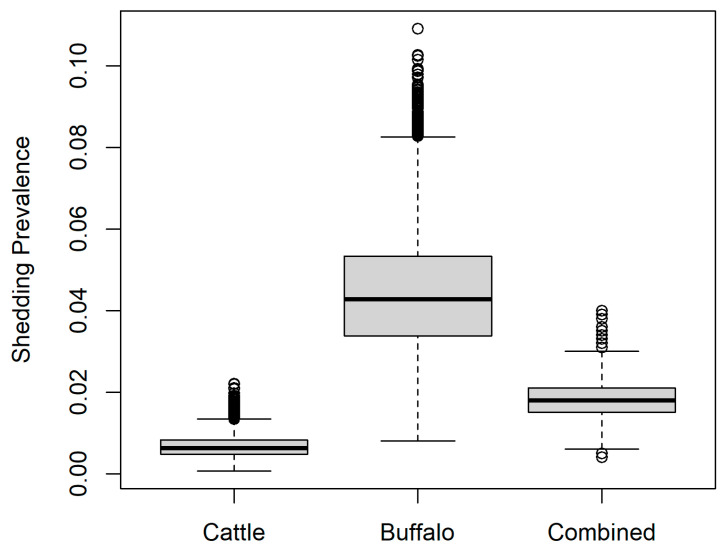
Estimated prevalence of shedding of *Brucella abortus* in the current study for buffalo (*Bubalis bubalis*), cattle (*Bos taurus*), and as a combined group according to the proportions of each species sampled in the current study.

Figure 2 shows the estimated prevalence of shedding of *B. abortus* (*Prev_shed_*; Equation (4)) for seropositive animals. For cows, this was mean 6.7 × 10^−3^ (median 6.3 × 10^−3^, 95% PI 2.5 × 10^−3^–13.2 × 10^−3^) according to the most conservative scenario (lowest proportion of seropositive cows that shed *B. abortus* according to a study in Iran [24]). For buffalo, the estimated prevalence of shedding of *B. abortus* was mean 0.04 (median 0.04, 95% PI 0.02–0.07) according to the most conservative scenario for buffalo in a study in Italy [23]. For both species combined in the proportions sampled in the current study, the overall estimated shedding prevalence was mean 1.8 × 10^−2^ (median 1.8 × 10^−2^, 95% PI 1.0 × 10^−2^–2.9 × 10^−2^).

**Figure 3:**
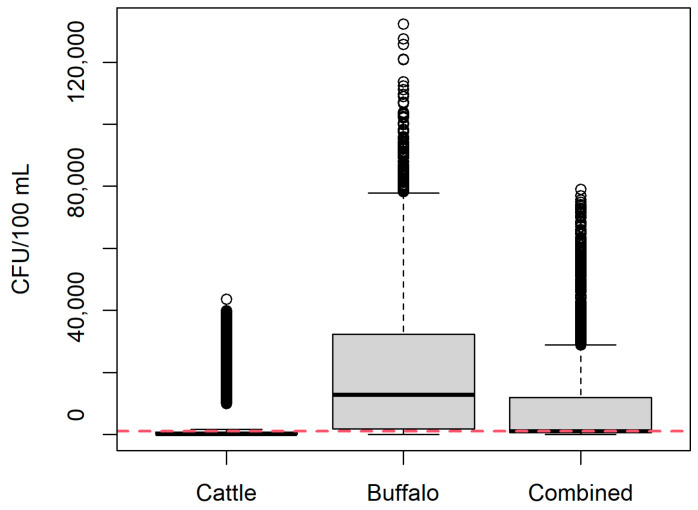
Estimated *Brucella abortus* CFU/100 mL unpasteurized milk in the current study for buffalo (*Bubalis bubalis*), cattle (*Bos taurus*), and as a combined group according to the proportions of each species sampled in the current study. Red line = 1000 CFU/100 mL.

Figure 3 shows the estimated CFU/100 mL of *B. abortus* in milk. For milk from cows (most conservative scenario), *B. abortus* CFU/100 mL was estimated as mean 2843 CFU/100 mL milk (median 240 CFU/100 mL; 95% PI 0–32,693 CFU/mL). For milk from buffalo (least conservative scenario), *B. abortus* CFU/100 mL was estimated as mean 17,963 CFU/100 mL milk (median 11,383 CFU/100 mL; 95% PI 612–67,121 CFU/mL). For milk from the combined group of cattle and buffalo representative of the current study, *B. abortus* CFU/100 mL was estimated as mean 7587 CFU/100 mL milk (median 956 CFU/100 mL; 95% PI 82–39,038 CFU/mL). For each scenario, the predicted probability of selecting 100 mL milk with >1000 CFU/mL was 0.09, 0.94, and 0.49 for cattle, buffalo and combined milk, respectively.

**Figure 4:**
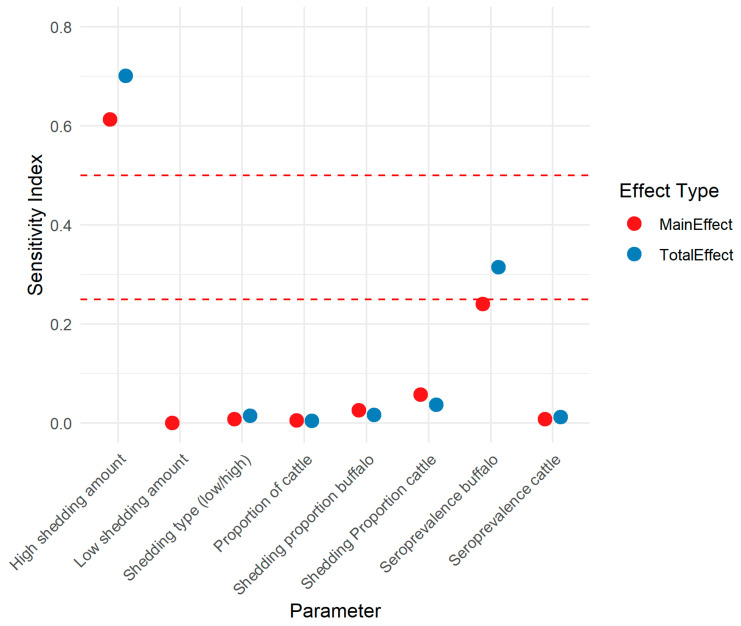
Sobol sensitivity indices for parameters in a risk assessment model of the amount of *Brucella abortus* colony forming units in milk sold via unregulated pathways (not pasteurized) in Punjab, India. Red dashed lines are at indices 0.25 and 0.5.

Figure 4 shows the main and total effect SIs for key input parameters. The number of CFU shed by high-shedding individuals was the most influential input parameter on the outcome, *B. abortus* CFU/100 mL (total effect 0.70; 95% CI 0.63–0.74), followed by seroprevalence in buffalo (total effect 0.29; 95% CI 0.25–0.33).

The model was re-run with high-shedding individuals removed, with and without infected buffalo. In the scenario with cattle only, *B. abortus* CFU/100 mL was estimated as mean 310 CFU/100 mL milk (median 192 CFU/100 mL; 95% PI 0–961 CFU/mL). In the scenario with buffalo and cattle, the *Brucella* CFU/100 mL was estimated as mean 842 CFU/100 mL milk (median 822 CFU/100 mL; 95% PI 62–1814 CFU/mL).

**Figure 5:**
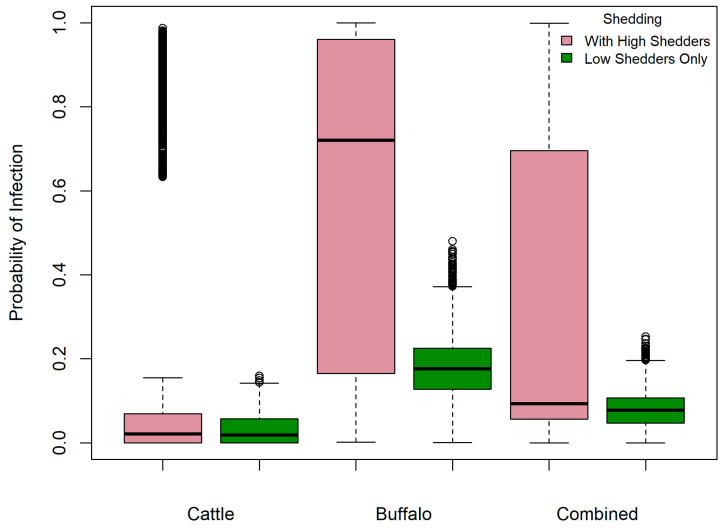
Probability of human infection with *Brucella abortus* given an infectious dose of 1000 CFU and consumption of unpasteurized 100 mL milk—with and without the inclusion of high-shedding bovines’ milk—in milk sold via unregulated pathways in Punjab, India.

Figure 5 shows the probability of human infection with *B. abortus*, given an infectious dose of 1000 CFU and consumption of 10 mL milk on a single occasion with and without the inclusion of high shedders. The probability for cattle-only, buffalo-only and combined milk scenarios was median 0.02 (95% PI 0–0.96) 0.72 (95% PI 0.06–0.99), and 0.09 (95% PI 0.01–0.98), respectively, and was reduced by 74%, 68%, and 73% for milk that was solely from low-shedding cattle, buffalo and combined species, respectively. However, a useful absolute reduction was only observed in the risk from buffalo milk (Table 3). If a person consumed 100 mL raw milk once monthly, their annual probability of *B. abortus* infection increased markedly, with median probability ≥0.20 for all scenarios and a maximum of only 9.8% absolute risk reduction when high shedders were removed, again in buffalo milk (Table 3).

## 4. Discussion

It is well known that the consumption of raw milk in India poses a significant risk of *Brucella abortus* infection in people. The additional information provided by this study is the influence of milk from high-shedding animals on this risk and the critical need for a One Health approach to prevention and control. Even given a low proportion of high shedders (estimated 17% in this study), a single consumption of 100 mL raw milk can pose a high risk of infection, but consumers who buy milk from unregulated sources do not always boil the milk prior to consumption. To mitigate this risk from unilateral perspectives, a public health approach would need to reduce the probability of raw milk consumption to zero, a regulatory approach would need to ban all sale of raw milk, and an animal health approach would need to eliminate *B. abortus* in cattle. These are all associated with enormous challenges.

Boiling or heating milk to at least 80–85 °C for several minutes destroys *Brucella* sp. bacteria [20], and successful education of consumers such that this is consistently conducted would eliminate the risk of brucellosis from milk. However, whilst education is important, it is unlikely to reach or be implemented by all target audiences, particularly those living in households that produce milk, or living rurally or in poverty with typically limited access to health messaging or the means to boil milk. Similar risks of exposure to *B. abortus* via milk have been identified in other regions. For example, in Tanzania, raw milk consumers had a high probability of *B. abortus* infection (0.64; 90% CI: 0.33–0.86), with an average daily consumption of 1.08 L (95% CI: 0.62–1.68 L [28]). Studies have also indicated that some population groups are more likely to consume raw milk, consistent with the lack of reach of messaging or feasibility of boiling milk in rural, farming, or low-income populations. For instance, in Côte d’Ivoire, 51.6% of residents in urban and peri-urban areas consumed raw milk, whilst in Tanzania, 71% of rural residents, yet only 10% of urban residents, reported consuming raw milk [28,29]. As well as location and socio-economic status, people’s occupations are also associated with brucellosis risk, likely due to additional risk pathways including exposure to animal birth products, but also increased access to raw milk. In Punjab, India, 69.6% of livestock farmers surveyed consumed raw milk [30]. Similarly, in Bihar, milk consumption was higher in households producing their own milk compared to those purchasing it from the market [31]. Nevertheless, even with increased advocacy to reach the coverage and effectiveness of health messaging and implementation of boiling milk to reduce raw milk consumption in vulnerable populations, the potential high risk from a single consumption of 100 mL raw milk makes additional mitigation measures essential.

Another avenue would be to strengthen regulations to reduce the sale of unpasteurized milk. As well as reducing the risk of infection with *B. abortus* from consuming raw milk, this could also reduce the risk of other foodborne infections. A recent meta-analysis reported a 14.6–45.8% prevalence of *Brucella* sp. contamination in dairy products in India including species such as *B. melitensis* (worldwide, the odds of co-contamination with *B. melitensis* [and *B. ovis*—although not a known pathogen in people] was reported as approximately two times higher than *B. abortus* alone) [32]). Unfortunately, this is also not straightforward to implement. Milk is an important food, and unregulated sources are typically cheaper than regulated sources. A ban or restriction on unregulated milk sales could reduce nutritional security, and cause economic harm to small dairy farmers who rely on it for their livelihoods or to those relying on employment in the dairy industry in rural areas where dairy farming is a major source of employment.

Ultimately, controlling brucellosis at the source in livestock is crucial for reducing transmission risks to people but this would be a very challenging, long-term goal. In cattle, the test-and-slaughter method is recommended for countries where individual animal prevalence is <2% [32]. In regions with higher prevalence such as India, vaccination of susceptible livestock (for example, *B. abortus* strain 19 and strain RB51 in cattle) and culling infected livestock is essential for control. Treating brucellosis in animals is discouraged–and banned in some countries, because bacteria can persist in tissues despite antibiotic treatment [32]. A key finding of the current study is that high-shedding individuals significantly influence the number of CFU/mL and hence the risk. Methods to identify and remove high-shedding animals would also be beneficial, although for smallholder or backyard producers, removal of animals through culling is not feasible, and discarding milk is not a financially attractive option. Since there is no human vaccine, this also highlights that using personal protective equipment (PPE) when handling animals—particularly during reproductive procedures—is crucial to reduce exposure risk [10]. However, missing the vaccination or removal of a high-shedding animal could undermine an entire control program, regardless of how comprehensive it is otherwise.

Therefore, given the constraints of unilateral sector controls, there is a need for a comprehensive, multi-sectorial control strategy that engages with all actors in the milk value chain, i.e., a One Health approach, to reduce the incidence and burden of brucellosis in India or other similar settings. Research to develop DIVA vaccines and improved *Brucella* diagnostics could underpin and improve farm-level controls (mandatory calf-hood vaccination and testing strategies to remove shedders–the most influential factor in this risk assessment), which would then provide a stronger foundation for village- to district-level controls such as surveillance (animal and human), improved animal health services to implement control strategies, and joint veterinary and public health education campaigns. These measures would generate critical background knowledge (e.g., identification of case hotspots) and establish control programs that could be supported by more targeted and enforceable regulations to reduce the sale of unpasteurized milk.

This study had limitations associated with assumptions about input parameters. The per capita milk availability is estimated at 1.23 L/day in Punjab [12], but the proportion of the population consuming raw milk and their average intake are not documented. However, a study in rural Bihar found that milk was consumed in 81% of surveyed households, with a median per capita intake of 83 mL/day (IQR: 11.9–166 mL/day) [31]. Given Punjab’s higher milk production, we consider that basing the risk estimate on consumption of 100 mL/day is reasonable, and given that approximately 70% of the milk produced in Punjab is sold through unregulated suppliers, such as informal milk shops and bicycle vendors, where pasteurization is typically absent [13]; our study demonstrates a significant likelihood of *B. abortus* infection through unregulated milk supply chains. Bovines can be infected with other *Brucella* species, such as *B. melitensis* and *B. suis* [33], which could further increase the risk of human exposure and subsequent brucellosis. Sensitivity analysis demonstrated that the presence of high shedders was most influential on risk. Unfortunately, the testing of milk in the current study was out of scope and the proportion of *B. abortus*-positive bovines shedding *Brucella* in milk was based on data from previous studies [23]. Whilst this might not fully reflect the livestock population in our study area, the variability in shedding levels is likely a biological phenomenon and we would expect similar variability in bovines in India. The prevalence of high shedders in the current study was relatively low, yet a high exposure risk to *B. abortus* remained, especially for buffalo-only milk. More place-based research needs to be conducted to identify if high shedders are associated with particular bovine species, because control strategies in bovines might need to be targeted to, for example, buffalo. Because data on the oral infectious dose for *B. abortus* were unavailable, we assumed it to be ten times higher than the highest estimated inhalation dose, providing a conservative estimate of risk. Lastly, we made the assumption that raw milk would be mixed from a variety of sources at the point of sale. It is likely that in reality, some vendors in the unregulated sector will source milk from farms with more or fewer high shedders than in the current study. Nevertheless, sensitivity analysis demonstrates that, given the influence of high shedders, the risk of brucellosis from raw milk consumption will be high regardless.

## 5. Conclusions

This study underscores that reducing the risk of brucellosis from milk consumption in Punjab, India, requires more than just livestock control measures—public health interventions are equally critical, because even a few high-shedding bovines, especially buffalo, can significantly increase the risk from raw milk consumption. A transdisciplinary approach that fosters collaboration between veterinarians, public health officials, and agricultural experts—i.e., a One Health approach—is essential to develop a comprehensive strategy for brucellosis prevention. However, all of the recommendations are challenging; strengthening milk safety regulations, enhancing public health campaigns to educate communities on the risks of consuming unpasteurized milk, and animal health measures all need to be implemented, and without any one of these, brucellosis will continue to pose a significant risk to vulnerable populations.

## Figures and Tables

**Table 1:** Results of serology testing for *B. abortus* antibody using the Rose Bengal plate test (RBPT) and indirect ELISA (I-ELISA) of lactating cows and buffalo sampled in Punjab, India.

Population	RBPT Positive (%)	I-ELISA Positive (%)	Both RBPT and I-ELISA Positive (%)	Total Tested
Cattle	35 (19.3)	45 (24.9)	33 (18.2)	181
Buffalo	11 (13.8)	13 (16.3)	8 (10.0)	80
Total	46 (17.6)	58 (22.2)	41 (15.7)	261

**Table 2:** Input data used in a risk assessment of the exposure to *Brucella* species after ingestion of cattle milk produced in Punjab, India.

Parameter	Name	Value	Distribution	References
Apparent seroprevalence of *Brucella abortus* in lactating cattle and buffalo	*Seroprevalence_apparent_*	mean 0.16 (95% range 0.12–0.21)	beta (42, 221)	Field data (current study)
Apparent seroprevalence of *Brucella abortus* in lactating cattle	*Seroprevalence_apparent_*	mean 0.19 (95% range 0.13–0.25)	beta (34, 149)	Field data (current study)
Apparent seroprevalence of *Brucella abortus* in lactating buffalo	*Seroprevalence_apparent_*	mean 0.11 (95% range 0.05–0.19)	beta (9, 73)	Field data (current study)
Probability of *Brucella* sp. shed in milk of seropositive cattle and buffalo—least conservative scenario (buffalo)	*P_shed_*	mean 0.30 (95% CI 0.25–0.35)	beta (102, 237)	[23]
Probability of *Brucella* sp. shed in milk of seropositive cattle and buffalo—most conservative scenario (cattle)	*P_shed_*	mean 0.03 (95% CI 0.01–0.05)	beta (7, 255)	[24]
Probability and shedding range (*Brucella* sp. CFU/mL) in low-shedding cattle and buffalo	*P_LowShed_*; *R_LowShed_*	mean 0.83 (95% range 0.75–0.9); 0–10^3^ CFU/mL	beta (86, 17); uniform (0, 1000)	[23]
Probability and shedding range (*Brucella* sp. CFU/mL) in high-shedding cattle and buffalo	1 − *P_LowShed_*; *R_HighShed_*	mean 0.17 (95% range 0.10–0.24); 1 × 10^4^–4 × 10^4^	1 − *P_LowShed_*; uniform (1 × 10^4^, 4 × 10^4)^	[23]

**Table 3:** Single meal and annual probability of human infection with *Brucella* abortus given an infectious dose of 1000 CFU and consumption of 100 mL milk once/month—with and without inclusion of high-shedding bovines’ milk—in milk sold via unregulated pathways (not pasteurized) in Punjab, India. NA = not applicable).

Population	Scenario	Median Single Consumption Risk (95% Predicted Interval [PI])	Absolute Risk Reduction (Single Consumption)	Median Annual Risk (95% PI)	Absolute Risk Reduction (Annual)
Cattle	With high shedders	0.02 (0–0.96)	NA	0.23 (0–1)	NA
Buffalo		0.72 (0.06–0.99)	NA	1.00 (0.55–1)	NA
Combined		0.09 (0.01–0.98)	NA	0.69 (0.10–1)	NA
Cattle	Only low shedders	0.02 (0–0.91)	0.2%	0.20 (0–0.68)	2.8%
Buffalo		0.18 (0.05–0.33)	54.4%	0.90 (0.46–0.99)	9.8%
Combined		0.08 (0.01–0.16)	1.5%	0.62 (0.07–0.88)	6.8%

## Data Availability

Data presented in this study are included in the article. Further inquiries can be directed to the corresponding author.
